# Early life trauma and endometriosis: A case-control study on type and timing of adverse experiences

**DOI:** 10.1007/s00737-026-01768-1

**Published:** 2026-09-19

**Authors:** Lauren Chiu, Eveline Mu, Qi Li, Lucy Ingleby, Jayashri Kulkarni

**Affiliations:** 1 Multidisciplinary Alfred Psychiatry Research Centre, Melbourne, Australia; 2 Department of Psychiatry, HER Centre Australia, Melbourne, Australia

**Keywords:** Endometriosis, Abuse, Trauma, Maltreatment, Prevalence

## Abstract

**Purpose:**

Endometriosis is a common chronic inflammatory gynaecological condition characterised by the abnormal presence of endometrial tissue outside the uterus. Different risk factors for endometriosis have been proposed, with some research finding a significant relationship between endometriosis and early life trauma. This study aimed to determine the prevalence, age, and type of early life trauma in a clinical sample of women with existing diagnosis of endometriosis compared with the prevalence in the general Australian female population.

**Methods:**

Participants were recruited from the Multidisciplinary Alfred Psychiatry research centre Women’s Mental Health Clinic (WMHC). Women who self-reported a diagnosis of endometriosis were identified from the WMHC patient database, established in 2017. In patients who reported a presence of early life trauma, information regarding the type and age of the early traumatic experience(s) was recorded. The prevalence of each type of trauma in our participants was compared to the general female population in Australia.

**Results:**

Of 68 included participants, the mean (SD) age was 41.6 (9.7) years. 86.8% of participants (*n* = 59) reported experience of any early life trauma, most commonly occurring between ages 6–10 (89.8%). The most reported type of trauma was emotional abuse (76.5%). Compared to the general female population, the prevalence of emotional abuse (76.5% vs. 37.3%), neglect (35.3% vs. 10.8%), and sexual abuse (39.7% vs. 37.3%) were higher in our sample.

**Conclusion:**

The findings of this study found high prevalence rates of early life trauma, in particular emotional abuse and neglect, in our clinical sample of women with endometriosis.

**Supplementary information:**

The online version contains supplementary material available at https://doi.org/10.1007/s00737-026-01768-1.

## Introduction

Endometriosis is a common chronic inflammatory gynaecological condition characterised by the abnormal presence of endometrial tissue outside the uterus, including on the pelvic peritoneum, ovaries, fallopian tubes, rectovaginal septum, and extrapelvic regions such as the gastrointestinal tract, skin, and central nervous system (Jubanyik and Comite 1997). Affecting both females and individuals assigned female at birth, there is an estimated 6–10% prevalence of pelvic endometriosis in this population (Houston 1984), increasing to 35–50% experiencing one or both symptoms of pain and infertility (Wheeler 1989). In addition to physical symptoms, emerging evidence identifies a high prevalence of comorbid mental illness, such as depression and anxiety, among individuals with endometriosis (Zippl et al. 2024).

The precise aetiology of endometriosis remains unclear, as well as an explanation as to why some women are affected by endometriosis while others are not. Current models theorise a ‘two-stage’ pathogenic process of endometriosis involving menstrual reflux as the initiating factor, followed by hormonal activity, impaired immunological response, and inflammation promoting the implantation of extrauterine endometrial cells (Parazzini et al. 2017). Different risk factors for endometriosis have also been proposed, with some recent research demonstrating a significant relationship between endometriosis and early life trauma (Harris et al. 2018; Liebermann et al. 2018).

The World Health Organization (WHO) classifies four types of childhood trauma: physical abuse, emotional abuse, sexual abuse, and neglect (Butchart 2006). Studies have previously established a link between exposure to childhood maltreatment and the development of physical disorders (Clemens et al. 2018), and in female populations, adverse childhood experiences are related to an increased risk of polycystic ovary syndrome (Mu et al. 2024), including correlation of sexual abuse with several other gynaecological issues including pelvic floor dysfunction, dyspareunia, and irregular menses (Vézina-Gagnon et al. 2018).

Research investigating the role of early life trauma in endometriosis is limited. In a large prospective cohort study (*N* = 60,595), Harris et al. (2018) found that the risk of laparoscopically-confirmed endometriosis was greater among women reporting severe physical or sexual abuse compared to those reporting no prior history, and a 79% increased risk of endometriosis for women who experienced chronic-severe abuse of multiple types. Liebermann et al. (2018) found the risk of endometriosis was greater among 842 participants with a history of emotional abuse, sexual abuse, neglect, and inconsistent experiences, with the greatest difference observed for emotional abuse. Conversely, of the different types of abuse, Tietjen et al. (2010) found a significant association only with physical abuse, with 15% of participants (*N* = 1,348) with endometriosis reporting exposure to physical abuse.

Dysfunction of the hypothalamic-pituitary-adrenal (HPA) axis is a hypothesised pathological mechanism linked to early life trauma (Westfall and Nemeroff 2015). The HPA axis is a dynamic feedback pathway that regulates cortisol secretion through a cascade of events triggered by stress responses in the body. In chronic stress, such as exposure to trauma, there is overactivity of the HPA axis and excess cortisol production as a consequence of altered central nervous system corticotropin-releasing hormone circuits (Heim et al. 2010). These enduring neuroendocrine changes may lead to the development of health complications such as autoimmune disease (Song et al. 2018), coronary heart disease (Wirtz and von Känel 2017), and polycystic ovarian syndrome (Mu et al. 2024). A study by Kuhlman et al. (2015) also found differences in physiological outcomes depending on the timing of trauma, with exposure to trauma during infancy related to acute stress dysregulation versus circadian cortisol dysregulation in later trauma exposure.

This case control study aims to address the limited research on the relationship between endometriosis and early life trauma, in a clinical mental health setting. We focus on evaluating the prevalence of childhood trauma in a clinical sample of women with existing diagnosis of endometriosis compared with the prevalence of early life trauma in the general Australian female population, and the relevance of type and age of early life trauma experience. Currently to our knowledge, few if any studies have been published that specifically explore the age of early life trauma experience. We hypothesise that in our clinical sample of women with mental ill health, the prevalence of early life trauma is higher in those with endometriosis than in the general female Australian population, and that certain ages and types of trauma experience are more prevalent than others, specifically childhood emotional abuse as suggested in existing literature (Gibb 2002).

## Materials and methods

### Participant recruitment

Participants were recruited from the Multidisciplinary Alfred Psychiatry research centre (MAPrc) Women's Mental Health Clinic (WMHC), a public service providing specialist secondary consultation for women referred with complex psychiatric concerns. Written informed consent was obtained from patients for their demographic and clinical information to be entered into the WMHC research database. Information on demographics, medical and psychiatric history, and retrospective developmental history, including experience of early life trauma, was collected via clinician-administered patient interviews.

### Data collection

The database was searched for all patients who attended the WMHC from 2017 to October 2024, and women who self-reported a diagnosis of endometriosis were identified. The experience of early life trauma, specifically neglect, emotional, physical or sexual abuse, was determined by experienced clinicians based on clinical interview. In patients who reported a presence of early life trauma, information regarding the type and age of the early traumatic experience(s) was recorded. Type of trauma was classified as: emotional abuse, physical abuse, neglect, or sexual abuse. Age of trauma experience was classified by fixed age groups: 0–5, 6–10, 11–14, and 15–18 years.

### Statistical analysis

Demographic and clinical variables were summarised using descriptive statistics. Frequency counts and proportions were used to calculate the overall prevalence of early life trauma within the sample of women with endometriosis, as well as the specific type of unique or co-occurring abuse experienced. The prevalence of emotional abuse, physical abuse, neglect, and sexual abuse in our participants was firstly compared to our total cohort of women with endometriosis, and secondly to the general female population in Australia, derived from the Australian Child Maltreatment Study (Mathews et al. 2023). Significance was determined using the χ^2^ statistic for categorical variables. The unadjusted odds ratio (OR) and accompanying 95% confidence interval (CI) were estimated for the types of trauma and age groups, with the 0–5 years group used as a reference group in all comparisons. OR was also employed in sensitivity analysis to explore the differences of prevalence across age groups in physical or mental health conditions. Statistical analysis was performed using R (Version 4.2.2, https://www.r-project.org/), and the figures were made in GraphPad Prism version 10.0 (GraphPad Software, Boston, Massachusetts USA, www.graphpad.com). Statistical significance was set at a *p* value of < 0.05.

## Results

### Participant characteristics

The demographic and clinical characteristics of our sample of women with endometriosis (*N* = 68) are summarised in Table 1. The mean age of participants was 41.6 years (SD = 9.68), with age range of 23–64 years.

**Table 1:** Demographic and clinical characteristics for women with endometriosis (*N* = 68)

	*N* (%)
Mean age in years [SD], range	41.6 [9.68], 23–64
Employment	
None/Studying	19 (27.9)
Casual	3 (4.4)
Part-time	16 (23.5)
Full-time	19 (27.9)
Missing	11 (16.2)
Highest education completed	
Year 11–12	5 (7.4)
Vocational training (TAFE, diploma)	13 (19.1)
Bachelor’s degree	23 (33.8)
Postgraduate degree	17 (25)
Missing	10 (14.7)
Alcohol use	
No current use	15 (22.1)
Infrequent or monthly	23 (33.8)
Daily or almost daily	9 (13.2)
Missing	21 (30.9)
Cigarette use	
Non-smoker	27 (39.7)
Current	6 (8.8)
Past smoker	14 (20.6)
Missing	21 (30.9)
Illicit substance use	
Never	23 (33.8)
Current	6 (8.8)
Past	18 (26.5)
Missing	21 (30.9)
Reproductive state	
Reproductive	29 (42.6)
Pregnant	4 (5.9)
Perimenopause	22 (32.4)
Post-menopause (natural)	5 (7.4)
Post-menopause (surgical)	8 (11.8)
Previous hysterectomy	11 (16.2)
Mental health diagnoses^†^	
Any	68 (100)
Major depressive disorder	43 (63.2)
Perimenopausal depression	22 (32.4)
Premenstrual dysphoric disorder	33 (48.5)
Generalised anxiety disorder	27 (39.7)
Complex trauma disorder	48 (70.6)
Other psychiatric condition	38 (55.9)
Physical health diagnoses^†^	
Hyperlipidaemia	10 (14.7)
Polycystic ovarian syndrome	13 (19.1)
Chronic fatigue syndrome	13 (19.1)
Fibromyalgia	13 (19.1)
Irritable bowel syndrome	10 (14.7)
Autoimmune condition	13 (19.1)
Other physical condition	42 (61.8)

^†^Some participants were diagnosed with >1 condition; N, number of participant; SD, standard deviation

The majority of women (42.6%) were in their reproductive years, followed by perimenopause (32.4%). Four participants (5.9%) were pregnant at the time of the clinic visit. Eleven participants (16.2%) had previously undergone a hysterectomy.

All women in our sample had received at least one mental health diagnosis currently or in the past, with the highest prevalences seen in complex trauma disorder (70.6%), major depressive disorder (63.2%), and premenstrual dysphoric disorder (48.5%). The most common comorbid physical health conditions were polycystic ovarian syndrome, chronic fatigue syndrome, fibromyalgia, and autoimmune disease (19.1%).

### Prevalence of early life trauma (type)

A high proportion of our sample of women with endometriosis experienced early life trauma, with 86.8% (*n* = 59) reporting a history of at least one of emotional abuse, physical abuse, neglect, or sexual abuse. Most women with a history of early life trauma experienced one type of trauma alone (35.3%), and emotional abuse was the type of trauma most commonly reported (76.5%). These findings are summarised in Table 2.

**Table 2:** Prevalences and ages of early life trauma (*N* = 68)

	Total, *N* (%)	Comparative test
Any experience of early life trauma^†^	59 (86.8)	
Number of types of trauma experienced^†^		
None	8 (11.8)	
1	24 (35.3)	
2	18 (26.5)	
> 3	18 (26.5)	
Age of abuse experienced (*N =* 59) ^†^		
0–5 years old	42 (71.2)	
6–10 years old	51 (86.4)	
11–14 years old	49 (83.1)	
15–18 years old	42 (71.2)	
Experience of trauma throughout childhood (all age groups 0–18 years old)	30 (44.1)	χ 2 = 6.3, ***p*** **= 0.012**
Emotional abuse	52 (76.5)	
0–5 years old	38 (73.1)	χ 2 = 23.6, ***p*** **< 0.001**
6–10 years old	46 (88.5)	χ 2 = 39.8, ***p*** **< 0.001**
11–14 years old	46 (88.5)	χ 2 = 39.8, ***p*** **< 0.001**
15–18 years old	37 (71.2)	χ 2 = 22.2, ***p*** **< 0.001**
Physical abuse	18 (26.5)	
0–5 years old	8 (44.4)	χ 2 = 21.1, ***p*** **< 0.001**
6–10 years old	13 (72.2)	χ 2 = 40.10, ***p*** **< 0.001**
11–14 years old	12 (66.7)	χ 2 = 36.02, ***p*** **< 0.001**
15–18 years old	9 (50)	χ 2 = 24.6, ***p*** **< 0.001**
Neglect	24 (35.3)	
0–5 years old	17 (70.8)	χ 2 = 37.9, ***p*** **< 0.001**
6–10 years old	20 (83.3)	χ 2 = 48.0, ***p*** **< 0.001**
11–14 years old	20 (83.3)	χ 2 = 48.0, ***p*** **< 0.001**
15–18 years old	19 (79.2)	χ 2 = 44.5, ***p*** **< 0.001**
Sexual abuse	27 (39.7)	
0–5 years old	8 (29.6)	χ 2 = 11.0, ***p*** **< 0.001**
6–10 years old	15 (55.6)	χ 2 = 26.1, ***p*** **< 0.001**
11–14 years old	11 (40.7)	χ 2 = 17.0, ***p*** **< 0.001**
15–18 years old	13 (48.1)	χ 2 = 21.39, ***p*** **< 0.001**

^†^Comparative analysis was not completed for *Any experience of early life trauma*, *Number of types of early life trauma experienced*, and *Age of abuse experienced*; *N*, number of participants; *p*, p value

### Prevalence of early life trauma (age)

**Fig. 1:**
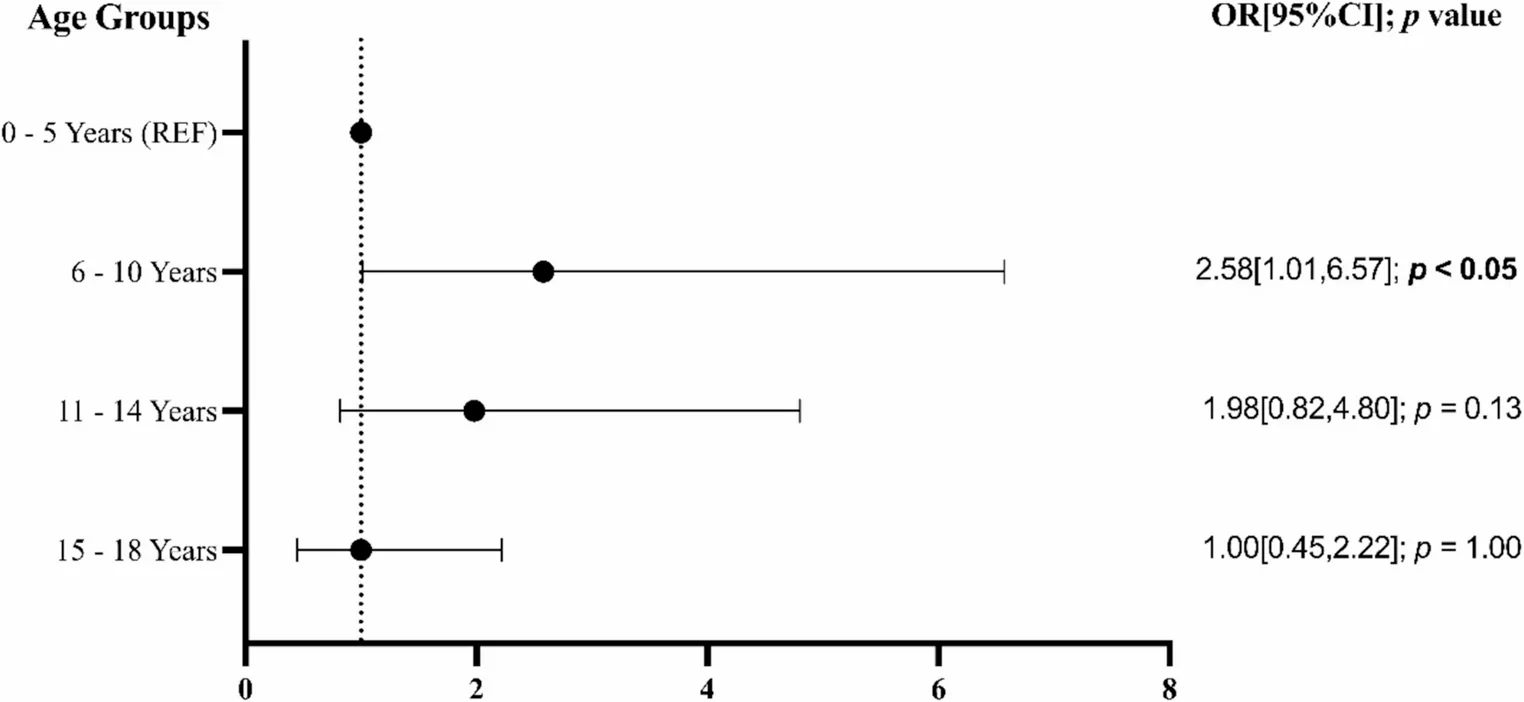
Forest plot comparing the prevalence of age groups at which early life trauma was experienced. OR, odds ratio; CI, confidence interval; REF, reference group for group comparison

**Fig. 2:**
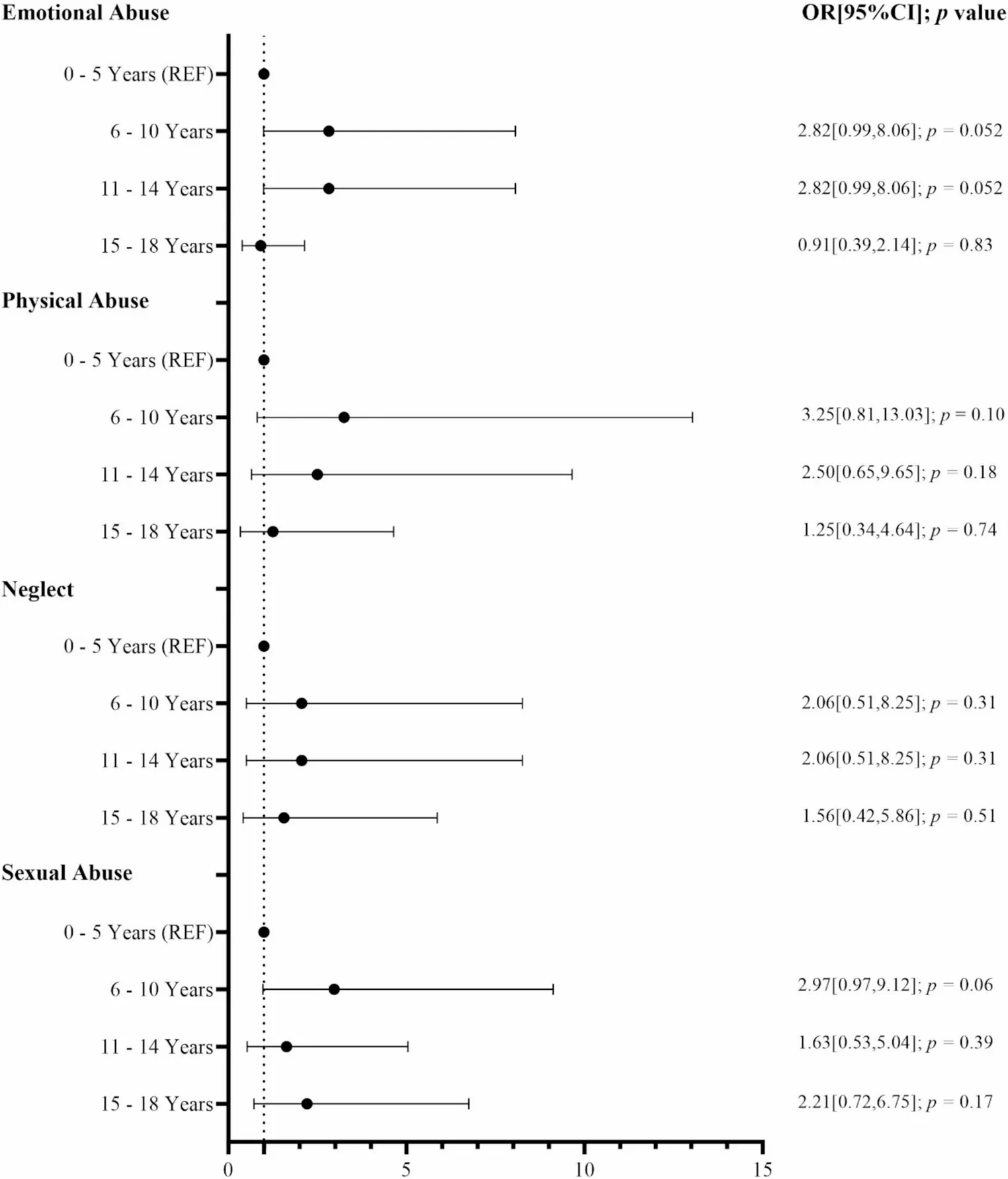
Forest plot comparing the prevalence of age groups by trauma type. OR. odds ratio; CI, confidence interval; REF, reference group for group comparison

Early life trauma was experienced throughout childhood across all four age groups (0–5, 6–10, 11–14, 15–18) for 44.1% of participants. 9 participants (13.2%) reported early life trauma limited within a single age category. Of the 59 participants reporting early life trauma, the age range at which this was most frequently experienced was 6–10 years of age (86.4%), closely followed by 11–14 years (83.1%). These findings are summarised in Figs. 1 and 2.

In the sensitivity analysis, the prevalence of emotional abuse in 6–10 years group and 11–14 years group were significantly higher than in the reference group (*p* = 0.03 and *p* = 0.03, respectively) in the population with generalized anxiety disorder as presented in the supplementary table (Online Resource 1).

### Comparison with general population

**Fig. 3:**
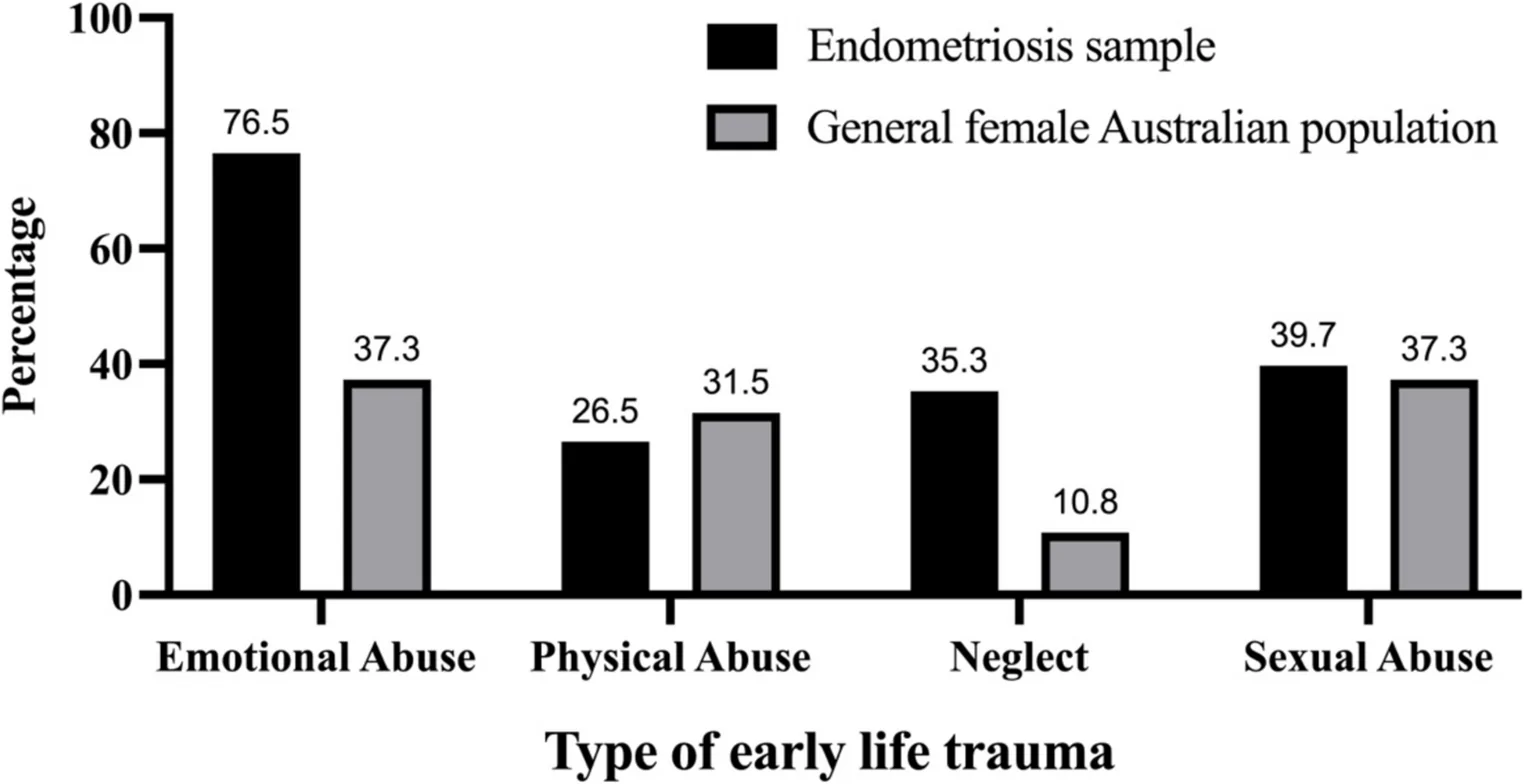
Prevalence of early life trauma experience by type, in women with endometriosis compared to the general female Australian population

The prevalence of early life trauma in our sample of women with endometriosis was greater compared to the general Australian female population (Mathews et al. 2023) for emotional abuse, sexual abuse, and neglect (Fig. 3).

**Fig. 4:**
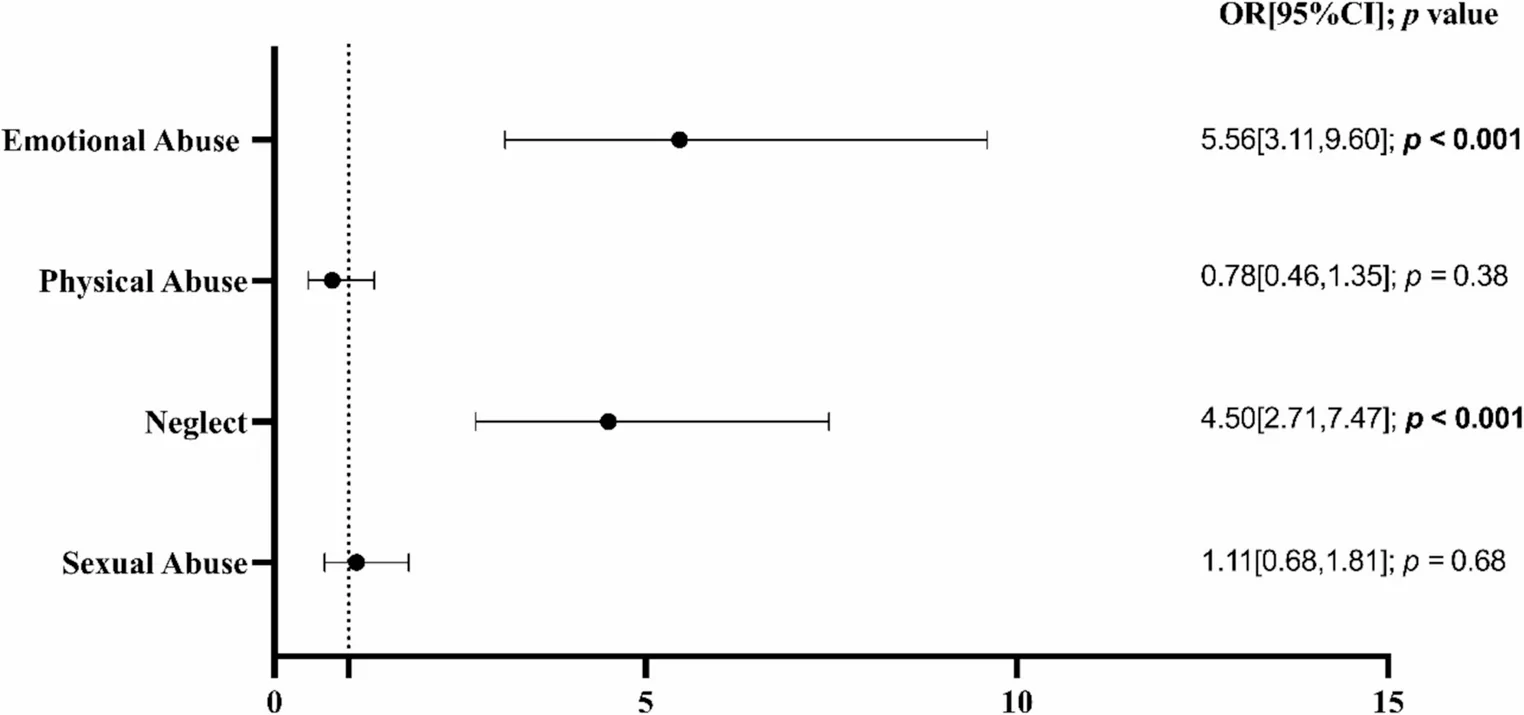
Forest plot comparing history of trauma in women with endometriosis to the general female Australian population, by type of trauma. OR, odds ratio; CI, confidence interval

Compared to the general population, the prevalences of emotional abuse and neglect were significantly greater in our sample by 39.2% (*p* < 0.001) and 24.5% (*p* < 0.001), respectively (Fig. 4). Sexual abuse was greater in our sample by 2.4% but not significantly different. Conversely, the prevalence of physical abuse was lower in our sample compared to the general female Australian population by 5%.

## Discussion

The primary aim of this study was to investigate whether the prevalence of childhood trauma in a clinical sample of women with endometriosis was higher compared to the general Australian female population. The secondary aim was to evaluate the relevance of age and type of early life trauma experience in this cohort.

### Prevalence of trauma

Our study found a high prevalence of trauma in women with endometriosis, with 86.8% of participants reporting experiencing childhood trauma. This finding is supported by the HPA axis model linking stress with excess cortisol production and neuroendocrine changes (Westfall and Nemeroff 2015). In particular, long-term consequences of repeated exposure to early life stress include downstream effects of HPA axis dysfunction, including immune dysregulation and compromised reproductive health, both pathophysiological features of endometriosis (Abramiuk et al. 2022; Neigh et al. 2009).

Past studies also support early life trauma as having a role in chronic inflammation (Grewal et al. 2024). Bertone-Johnson et al. (2014) found that women who reported childhood sexual abuse had higher plasma levels of interleukin-6 and C-reactive protein than those who did not, suggesting that early life abuse may contribute to chronic inflammation through dysregulated immune function. Moreover, Kerr et al. (2021) established a significant association between early life trauma and increased inflammation when compared to controls without past exposure to trauma. This is potentially important when considering inflammation as a pathogenic hallmark of endometriosis, explained by mechanisms of peritoneal macrophage infiltration and local proinflammatory mediators (Parazzini et al. 2017; Wu et al. 2015). Epidemiologically, these findings may be linked to the overall comparatively higher prevalence of early life trauma observed in our endometriosis sample.

### Type of trauma

Our results showed prevalence rates of different types of childhood trauma ranging from 26.5% to 76.5%. There was a significantly higher prevalence of early life emotional abuse and neglect in women with endometriosis compared to the general female population, suggesting that women with endometriosis are more likely to have experienced these specific types of early life trauma. Our findings are consistent with Liebermann et al. (2018), who found that childhood emotional abuse and neglect were significantly associated with endometriosis. The lower prevalences of physical and sexual abuse observed in our cohort are also in line with studies by Schliep et al. (2016), however, they contrast with the findings of Tietjen et al. (2010), who found significant associations with physical abuse, sexual abuse, and emotional abuse.

The significant downstream impacts following emotional abuse and neglect in early life may be due to a variety of mechanisms. Existing literature has suggested that childhood emotional abuse is the most harmful type of early life trauma for children (Gibb 2002). In these instances, this may be due to the caregiving role the perpetrator typically holds in relation to the victim, which involves a violation of the love and respect usually expected (Kulkarni et al. 2022). Emotional abuse also tends to be more verbal, enabling greater internalisation of negative self-perceptions in the victim (Sachs-Ericsson et al. 2006).

### Age of trauma

Overall, across all types of trauma, childhood trauma occurring between the ages of 6–10 and 16–14 was more common than other age groups at prevalences of 86.4% and 83.1%, respectively. This may reflect the age-dependent neurobiological developmental processes in which rapid brain changes are observed in specific developmentally sensitive periods of childhood (Thomas et al. 2019). Trauma in early life may also be more harmful than trauma experienced later in life, as it interferes with a child’s ability to effectively navigate critical developmental milestones, such as forming secure attachments and self-regulation (Dunn et al. 2017). Similarly, Jean Piaget’s theory of cognitive development also represents middle childhood as a period for shifts in egocentrism and mentalisation (Piaget 1971). Disruptions to cognitive and social development during this important period, such as through adverse childhood experiences, may lead to long-term psychological difficulties in self-concept, emotional regulation, and interpersonal functioning (National Research Council Panel to Review the Status of Basic Research on School-Age 1984).

We found that 44.1% of women experienced early life trauma throughout childhood, between the ages 0–18. This finding reflects a high prevalence of chronic early life trauma in our clinical sample of women with endometriosis. In comparison, only 13.2% of participants experienced trauma within an isolated age group. This may suggest that prolonged exposure to trauma throughout childhood is more strongly associated with endometriosis rather than a specific age of exposure.

### Limitations and implications

Several limitations need to be taken into consideration. Firstly, there was inherent sampling bias in our participant group, given our participants were recruited from a specialist consultation clinic for women with complex psychiatric concerns, therefore impacting generalisability to the general female population. For this reason, it is also possible that our participants were more likely to report history of early life trauma given they were recruited from a mental health clinic.

The data from this study were obtained cross-sectionally from participants’ retrospective self-reports, thus enabling a degree of recall bias. As such, it is possible that childhood trauma occurring in the 0–5 age group was also underreported. Moreover, although trauma history was ascertained through clinical interview by experienced clinicians, this was not carried out through standardised methods of assessing trauma, for example, through a validated questionnaire or survey. However, it should be noted that there has been a high convergent validity between self-report questionnaires and comprehensive interviews (Gayer-Anderson et al. 2020).

Finally, there was no healthy control group to compare trauma characteristics and prevalence with our endometriosis sample. Instead, prevalence figures of the general female Australian population were utilised as a point of comparison. As a result, descriptive comparisons of participant demographic characteristics and age of trauma were unable to be undertaken.

While some previous research has investigated the prevalence of trauma in individuals with endometriosis (Harris et al. 2018; Liebermann et al. 2018; Tietjen et al. 2010), to our knowledge, this is the first study to explore the ages at which trauma was experienced. Our study also indicates a need for further research investigating emotional abuse and neglect specifically, given these types of trauma are generally less studied than physical and sexual abuse (Harris et al. 2018; Schliep et al. 2016; Tietjen et al. 2010). Our findings suggest a significant difference may exist between certain types and ages of childhood trauma and the development of endometriosis. By identifying high prevalence rates of early life trauma in women with endometriosis, our findings support the importance of assessing prior trauma when obtaining a comprehensive clinical history in patients with endometriosis. Likewise, targeted screening for trauma and specific abuse profiles may assist clinicians in identifying individuals at higher risk of developing endometriosis. A focus on effective prevention and intervention of early life trauma is likely essential to mitigate the many long-term health consequences and other downstream effects of trauma that can arise.

## Conclusion

Our cohort of women with endometriosis had a significantly higher prevalence of early life emotional abuse and neglect compared to the general Australian population. Additionally, prolonged exposure to trauma throughout childhood may increase the risk of developing endometriosis. Our study contributes to understandings of the relationship between early life trauma and endometriosis. More research is necessary to further characterise this relationship in future studies.

## Supplementary information

Below is the link to the electronic supplementary material.

Supplementary File 1 (PDF 97.4 KB)

## Data Availability

No datasets were generated or analysed during the current study.
