# Supplementary material for: Early life trauma and endometriosis: A case-control study on type and timing of adverse experiences
Source: Arch Womens Ment Health. 2026 Sep 19;29(5):127. doi: 10.1007/s00737-026-01768-1 (PMC13588869; doi:10.1007/s00737-026-01768-1)
Supplement: Supplementary file 1 [file 737_2026_1768_MOESM1_ESM.pdf]

Supplemental Information

Table 1 Statistical analysis for comorbid physical and mental health conditions

Sample size = 43

**Major depressive disorder**	**Emotional abuse**	**Physical abuse**	**Neglect**	**Sexual abuse**
0 – 5 years old	1.00	1.00	1.00	1.00
6 – 10 years old	1.72 [0.69, 4.34]	1.18 [0.39, 3.59]	1.35 [0.56, 3.28]	1.36 [0.46, 4.06]
11 – 14 years old	1.53 [0.62, 3.80]	1.36 [0.46, 4.06]	1.11 [0.45, 2.71]	1.00 [0.32, 3.14]
15 – 18 years old	0.75 [0.32, 1.77]	1.00 [0.32, 3.14]	0.90 [0.36, 2.23]	1.00 [0.32, 3.14]

Sample size = 22

**Perimenopausal depression**	**Emotional abuse**	**Physical abuse**	**Neglect**	**Sexual abuse**
0 – 5 years old	1.00	1.00	1.00	1.00
6 – 10 years old	2.14 [0.63, 7.30]	2.38 [0.51, 11.05]	1.59 [0.41, 6.07]	2.38 [0.51, 11.05]
11 – 14 years old	2.14 [0.63, 7.30]	1.41 [0.28, 7.18]	1.59 [0.41, 6.07]	1.86 [0.39, 8.99]
15 – 18 years old	2.14 [0.63, 7.30]	1.41 [0.28, 7.18]	1.59 [0.41, 6.07]	1.41 [0.28, 7.18]

Sample size = 33

**PMDD**	**Emotional abuse**	**Physical abuse**	**Neglect**	**Sexual abuse**
0 – 5 years old	1.00	1.00	1.00	1.00
6 – 10 years old	1.67 [0.62, 4.52]	2.77 [0.50, 15.42]	1.15 [0.41, 3.24]	3.44 [0.64, 18.51]
11 – 14 years old	1.92 [0.70, 5.26]	2.77 [0.50, 15.42]	1.31 [0.47, 3.67]	1.55 [0.24, 9.94]
15 – 18 years old	1.13 [0.43, 2.99]	2.14 [0.36, 12.57]	1.50 [0.54, 4.14]	3.44 [0.64, 18.51]

Sample size = 27

**GAD**	**Emotional abuse**	**Physical abuse**	**Neglect**	**Sexual abuse**
0 – 5 years old	1.00	1.00	1.00	1.00
6 – 10 years old	**6.25 [1.20, 32.47] * (*p* = 0.03)**	2.01 [0.51, 7.90]	1.68 [0.53, 5.37]	2.01 [0.51, 7.90]
11 – 14 years old	**6.25 [1.20, 32.47] * (*p* = 0.03)**	2.42 [0.63, 9.30]	1.43 [0.44, 4.63]	1.64 [0.41, 6.64]

15 – 18 years old	1.43 [0.44, 4.63]	1.64 [0.41, 6.64]	1.43 [0.44, 4.63]	2.01 [0.51, 7.90]

Sample size = 48

**CPTSD**	**Emotional abuse**	**Physical abuse**	**Neglect**	**Sexual abuse**
0 – 5 years old	1.00	1.00	1.00	1.00
6 – 10 years old	2.02 [0.83, 4.92]	2.08 [0.70, 6.18]	1.31 [0.57, 3.02]	2.06 [0.77, 5.49]
11 – 14 years old	1.80 [0.75, 4.32]	1.62 [0.53, 4.96]	1.31 [0.57, 3.02]	1.32 [0.47, 3.69]
15 – 18 years old	1.09 [0.48, 2.51]	1.20 [0.37, 3.86]	1.20 [0.52, 2.77]	1.67 [0.61, 4.54]

Sample size = 51

**Other mental health condition**	**Emotional abuse**	**Physical abuse**	**Neglect**	**Sexual abuse**
0 – 5 years old	1.00	1.00	1.00	1.00
6 – 10 years old	1.63 [0.74, 3.61]	2.13 [0.50, 9.04]	1.26 [0.49, 3.25]	1.46 [0.43, 4.96]
11 – 14 years old	1.39 [0.63, 3.09]	2.98 [0.74, 11.94]	1.40 [0.55, 3.57]	1.23 [0.35, 4.31]
15 – 18 years old	1.28 [0.58, 2.86]	1.74 [0.39, 7.70]	1.26 [0.49, 3.25]	1.46 [0.43, 4.96]

Sample size = 10

Hyperlipidaemia	Emotional abuse	Physical abuse	Neglect	Sexual abuse
0 – 5 years old				
6 – 10 years old	1.00 [0.17, 5.77]	1.00 [0.02, 55.27]	1.00 [0.15, 6.77]	1.00 [0.02, 55.27]
11 – 14 years old	1.50 [0.26, 8.82]	3.32 [0.12, 91.61]	1.00 [0.15, 6.77]	1.00 [0.02, 55.27]
15 – 18 years old	1.00 [0.17, 5.77]	1.00 [0.02, 55.27]	1.00 [0.15, 6.77]	1.00 [0.02, 55.27]

Sample size = 13

PCOS	Emotional abuse	Physical abuse	Neglect	Sexual abuse
0 – 5 years old				
6 – 10 years old	2.85 [0.53, 15.47]	1.65 [0.23, 11.99]	1.48 [0.26, 8.50]	2.44 [0.36, 16.55]
11 – 14 years old	2.85 [0.53, 15.47]	2.44 [0.36, 16.55]	2.08 [0.38, 11.48]	1.00 [0.12, 8.42]
15 – 18 years old	1.92 [0.39, 9.60]	1.00 [0.12, 8.42]	1.48 [0.26, 8.50]	1.00 [0.12, 8.42]

Sample size = 13

Chronic fatigue syndrome	Emotional abuse	Physical abuse	Neglect	Sexual abuse
0 – 5 years old				
6 – 10 years old	0.68 [0.12, 3.87]	1.00 [0.16, 6.20]	1.00 [0.16, 6.20]	1.00 [0.16, 6.20]
11 – 14 years old	1.00 [0.16, 6.20]	1.00 [0.16, 6.20]	0.61 [0.08, 4.41]	0.61 [0.08, 4.41]
15 – 18 years old	0.26 [0.05, 1.39]	0.28 [0.02, 3.10]	0.28 [0.02, 3.10]	0.61 [0.08, 4.41]

Sample size = 13

Fibromyalgia	Emotional abuse	Physical abuse	Neglect	Sexual abuse
0 – 5 years old				
6 – 10 years old	1.48 [0.26, 8.50]	1.41 [0.28, 7.13]	1.00 [0.21, 4.86]	2.08 [0.38, 11.48]
11 – 14 years old	2.44 [0.36, 16.55]	1.41 [0.28, 7.13]	0.71 [0.14, 3.61]	1.48 [0.26, 8.50]
15 – 18 years old	0.71 [0.14, 3.61]	1.00 [0.19, 5.29]	0.48 [0.09, 2.65]	1.48 [0.26, 8.50]

Sample size = 10

IBS	Emotional abuse	Physical abuse	Neglect	Sexual abuse
0 – 5 years old				
6 – 10 years old	2.25 [0.17, 29.77]	2.33 [0.37, 14.61]	1.71 [0.22, 13.41]	1.71 [0.22, 13.41]
11 – 14 years old	1.00 [0.11, 8.95]	1.56 [0.24, 9.91]	1.00 [0.11, 8.95]	2.67 [0.36, 19.71]
15 – 18 years old	0.58 [0.07, 4.56]	1.00 [0.15, 6.77]	1.00 [0.11, 8.95]	4.00 [0.55, 29.10]

Sample size = 13

Autoimmune condition	Emotional abuse	Physical abuse	Neglect	Sexual abuse
0 – 5 years old				
6 – 10 years old	1.93 [0.39, 9.60]	3.60 [0.32, 40.23]	1.00 [0.12, 8.42]	3.60 [0.32, 40.23]
11 – 14 years old	2.86 [0.53, 15.47]	1.00 [0.06, 17.90]	1.00 [0.12, 8.42]	1.00 [0.06, 17.90]
15 – 18 years old	1.93 [0.39, 9.60]	2.18 [0.17, 27.56]	1.65 [0.23, 11.99]	2.18 [0.17, 27.56]

Sample size = 44

Other physical health condition	Emotional abuse	Physical abuse	Neglect	Sexual abuse
0 – 5 years old				
6 – 10 years old	1.59 [0.68, 3.71]	2.57 [0.73, 9.09]	1.26 [0.49, 3.22]	2.94 [0.85, 10.23]
11 – 14 years old	1.59 [0.68, 3.71]	1.89 [0.51, 6.99]	1.26 [0.49, 3.22]	1.58 [0.41, 6.04]
15 – 18 years old	1.10 [0.47, 2.53]	1.58 [0.41, 6.04]	1.26 [0.49, 3.22]	1.28 [0.32, 5.13]
